# Active Biomolecules from Vegetable Extracts with Antitumoral Activity against Pancreas Cancer: A Systematic Review (2011–2021)

**DOI:** 10.3390/life12111765

**Published:** 2022-11-02

**Authors:** Cristina Mesas, Francisco Quiñonero, Kevin Doello, José L. Revueltas, Gloria Perazzoli, Laura Cabeza, Jose Prados, Consolación Melguizo

**Affiliations:** 1 Institute of Biopathology and Regenerative Medicine (IBIMER), Center of Biomedical Research (CIBM), University of Granada, 18100 Granada, Spain; 2 Department of Anatomy and Embryology, Faculty of Medicine, University of Granada, 18071 Granada, Spain; 3 Instituto Biosanitario de Granada (ibs. GRANADA), 18014 Granada, Spain; 4 Medical Oncology Service, Virgen de las Nieves Hospital, 18016 Granada, Spain; 5 Radiodiagnosis Service, Reina Sofía University Hospital, 14004 Córdoba, Spain

**Keywords:** pancreatic cancer, plant extract, biomolecule, drug resistance, antitumor activity

## Abstract

The emergence of resistance to pancreatic cancer (PC) current treatment requires the development of new therapeutic strategies. In this context, bioactive molecules from plant extracts have shown excellent properties to improve classical therapy against this type of tumor. This systematic review aims to collect all the in vitro studies related to the antiproliferative activity of isolated plant molecules that support their applicability in PC. A total of 620 articles published in the last 10 years were identified, although only 28 were finally included to meet the inclusion criteria. Our results reflect the most important biomolecules from natural compounds that induce cell death in PC and their essential mechanism of cell death, including apoptosis, pathways activated by the KRAS mutation and cycle cell arrest, among others. These in vitro studies provide an excellent molecule guide showing applications against PC and that should be tested in vivo and in clinical trials to determine their usefulness to reduce PC incidence and to improve the prognosis of these patients. However, natural compounds are isolated in small amounts, which prevents comprehensive drug screening, being necessary the role of organic synthesis for the total synthesis of natural compounds or for the synthesis of their simplified and bioactive analogs.

## 1. Introduction

Pancreatic cancer is the fourth leading cause of cancer death in the United States and Europe. One of the major problems with this type of tumor is the lack of effective therapy, with only 20% of tumors resectable by surgery since the mass is localized and in the early stages. However, most tumors are in more advanced stages when diagnosed, and these are often metastatic [1]. This absence of an early diagnosis causes PC to be one of the tumors with a survival rate of less than 10%, and may even be around 4–5%, occupying the seventh place in terms of rate of fatality worldwide [2]. Surgical resection, the only treatment capable of curing PC, is often supplemented by adjuvant chemotherapy with gemcitabine or a combination of drugs called FOLFIRINOX. For their part, metastatic patients are treated only with the gemcitabine or FOLFIRINOX regimen (combined with 5-fluorouracil), although a very low survival time is achieved (between 11 and 7 months). Therefore, it is necessary to discover new therapies capable of increasing the quality of life of patients and ideally that are capable of causing tumor resection [3].

In recent years, the use of natural products in various areas related to cancer, such as preventive diets or complementary therapy, has gained great importance [4,5]. Due to the limitations of current therapies, the therapeutic approach to cancer using biomolecules from natural products has great relevance, especially as supplementary therapies. As advantages derived from the use of these biomolecules, it could be highlighted their low level of toxicity and the possibility of enhancing the anticancer effect of the drugs used while reducing their toxicity [6]. Among the different groups of natural products with anticancer action, polyphenols, cardiac glycosides, terpenoids and saponins, among others, are the most relevant [7]. Polyphenols, a group of chemical substances found in fruits, cereals and vegetables, have demonstrated anti-inflammatory and anticancer effects [8,9,10,11,12]. Polyphenols include the flavonoid family, such as flavonoids (apigenin or wogonin), flavans, flavonols and flavanones, and the nonflavonoid family, which includes curcumin and resveratrol as major members [7]. In addition, glycosides, traditionally used as an antiarrhythmic and in heart failure treatment, have been also assayed in cancer. In fact, digoxin, among other drugs in this group, could have antitumoral effects that could give a new approach to its use [7,13,14,15,16]. In addition, terpenoids, compounds derived from mevalonic acid, have demonstrated anti-inflammatory, antineoplastic and antibacterial effects. Paclitaxel, a clear example of these molecules, has been widely used as an antitumor in breast or lung cancer [7,17,18,19]. Finally, saponins, like the previous ones, exhibit important analgesic, anti-inflammatory and antitumor effects. These molecules have been explored as adjuvant therapy or to develop antitumor vaccines such as ISCOMATRIX, which activate the immune response of B and T lymphocytes [7,20,21].

In this systematic review, different plant families will be analyzed from which functional extracts with antitumor activity against PC are obtained or from which compounds with antitumor activity can be isolated that can be used as a possible therapy and adjuvant in the current treatment of PC.

## 2. Materials and Methods

### 2.1. Study Eligibility

The purpose of this systematic review was to assess the most recent and representative information on extracts and natural biomolecules with antitumor activity against PC. This systematic review was developed according to the PRISMA guide [22]. For this, the bibliometric analysis was carried out for the last 10 years (2011–2021), considering older studies obsolete. Thus, most of the current articles published on this topic were included according to the Burton–Kebler obsolescence index based on middle age/medium production [23].

### 2.2. Inclusion Criteria

Research papers from March 2011 to March 2021, which included isolated biomolecules or plant extracts tested on PC cell lines, were incorporated in this systematic review. In addition, the studies had to include IC_50_ values and specify the mechanism of action by which the plant extracts or isolated compounds exert their antitumor power. All the research articles had been published in peer-reviewed journals, and those with fully accessible texts were selected. Articles found through bibliographic references could also be included, as long as they met the inclusion criteria.

### 2.3. Exclusion Criteria

Studies that did not test the extracts or compounds isolated in PC cells, those in which the biomolecules or extracts were synthesized or purchased, that did not specify an extraction method, that the article was not freely accessible or that were written in Chinese or Japanese were excluded. Articles that studied extracts or compounds from other kingdoms than plantae were also discarded. In addition, articles that only included IC_50_ or only explained the anticancer mechanism of action, without providing IC_50_ values, were excluded. In turn, nonoriginal articles, such as reviews, meta-analyses, epidemiological studies, book chapters or conferences, were also excluded from the study.

### 2.4. Data Sources

This systematic review was carried out using the electronic databases: MedLars Online International Literature, through PubMed; SCOPUS; Web of Science; and Cochrane Library Plus. The medical subject headings (MeSH) were defined using “pancreatic neoplasm” and “plant extract” as descriptive terms. The final equation was ((“Pancreatic Neoplasms” [MeSH Terms] OR (“pancreatic” [Title/Abstract] AND “neoplasms” [Title/Abstract]) OR “Pancreatic Neoplasms” [Title/Abstract] OR “pancreatic cancer” [Title/Abstract]) AND (“Plant Extracts” [MeSH Terms] OR “Plant Extracts” [Title/Abstract])) AND (2011/3/1:2021/3/1 [pdat]). For the other databases, adaptations were made to carry out the search. In addition, the bibliography of the selected articles was reviewed to analyze if they met the inclusion criteria.

### 2.5. Study Selection

Three of the authors (C.M., F.Q. and J.L.R.) carried out the literature search, the review of the abstracts and the selection of the appropriate ones for further full-text examination. In turn, nonoriginal articles, such as reviews, meta-analyses, epidemiological studies, book chapters or conferences, were also discarded from the study. There were restrictions by language, excluding those who were in a language other than English or Spanish. After this first step, the author examined the full-text articles, considering the inclusion and exclusion criteria. Since the objective of this systematic review was to review in vitro studies, those where only in vivo studies were performed were excluded.

### 2.6. Data Extraction

**Figure 1:**
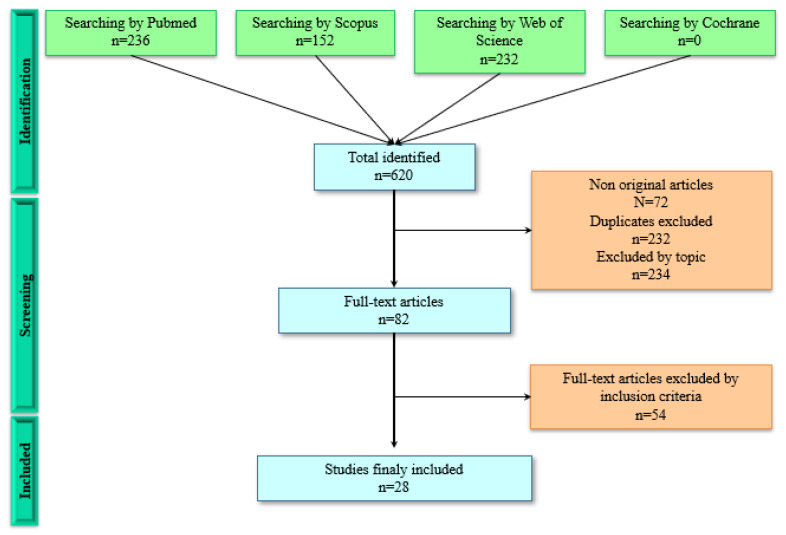
Flow diagram in which the methodology carried out is represented.

To continue the study selection process, the same three authors independently reviewed the studies included and extracted data from them. There was good agreement between the researchers [24] according to the Cohen Kappa statistical test [25], which exceeded 0.8. In the process, any discrepancies were resolved between authors C.M., F.Q., and J.L.R. If a consensus was not reached, authors J.P. and C.M.A. would intervene if necessary. A specific questionnaire for in vitro model studies was used to determine the quality of the article included. The questionnaire consists of a first part to determine the premises of an in vitro study (score >6) and a second part to determine the quality of the study (0–6¼, low; 7–14¼, good; 15–20¼, excellent), including the materials and methods, results, and conclusions. Table 1 summarizes the data extracted from the studies included. Data were classified according to their family, specie and mechanism of action. In addition, the number of articles, the plant part that was used to make the extract and the mechanism of action by which they induce cell death were indicated. As can be seen in Figure 1, 620 articles derived from the selected databases were obtained. After discarding the nonoriginal articles (72), duplicates (232) and excluded by topic (234), 82 articles were selected. Following the inclusion and exclusion criteria, 28 articles were finally obtained.

## 3. Results

**Figure 2:**
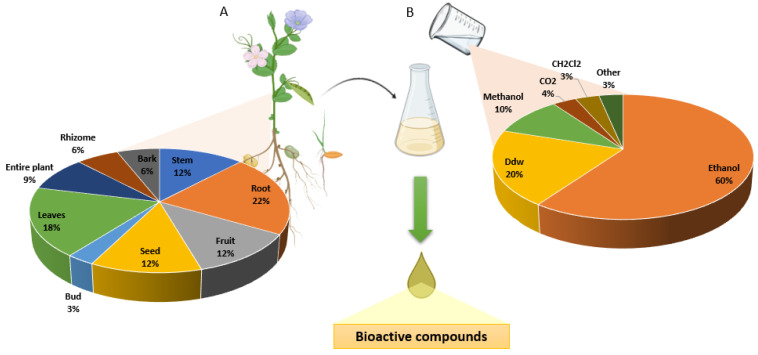
Representation of the plant part most used to obtain functional extracts (**A**) and the type of solvent most used to carry it out (**B**).

The results were presented considering the main mechanism by which natural compounds induce cell death in PC cell lines. Of the articles included, 11 presented functional extracts or isolated compounds that showed antitumor activity through apoptosis, 5 described functional extracts that altered the pathways activated by the mutation in the *KRAS* gene, 7 demonstrated cell death due to cell cycle arrest cell and 4 other articles describe other actions through important factors in PC. The most frequent part of the plant used to obtain the bioactive compounds were the roots, followed by the leaves, stems, fruits and seeds. The shoots and bark were less studied (Figure 2A). On the other hand, the extraction process of the bioactive compounds included in most cases ethanol as solvent followed by distilled water and methanol (Figure 2B).

### 3.1. Plant Species and Isolated Compounds That Induce Cell Death by Apoptosis

Table 2 shows plant species and isolated compounds that induce cell death by apoptosis. The antitumor activity of *Oplopanax horridus* (Sm.) Miq. was analyzed in two studies. In the first, ethanol and distilled water were used to obtain the plant stem extracts. Then, liquid chromatography (HPLC) was used to obtain four preparations of the active compound DCA. The IC_50_ for the different compounds ranged between 0.22 and 5.5 µg/mL on the PANC-1 pancreatic cell line. The value found for the distilled water extract was negligible. On the BxPC-3 cell line, the values varied between 0.82 and 34 µg/mL, without having tested the aqueous extract. In the different DCA preparations, only 1 and 3 showed activity. The DCA and ethanolic extracts showed more potency than the aqueous extract. It was observed that the products of *O. horridus* produced an increase in the number of nuclear apoptotic bodies, visualized with fluorescence. In turn, the effect of DCA on 35 genes associated with apoptosis was studied, suggesting cell death by apoptosis through the extrinsic and intrinsic pathways [26]. In the second article, Cheun et al. [27] used the dried roots of the plant to obtain an extract of ethanol and DCA, also by using HPLC. This extract showed an IC_50_ of 5.8 and 21 nM in the PANC-1 and BxPC-3 pancreatic cell lines, respectively. The DCA, for its part, showed an IC_50_ of 0.73 and 2.71 µM in PANC-1 and BxPC-3 cells, respectively. Apoptosis mechanism was mediated by an increase in caspase-3 and cytochrome C in the cell cytoplasm and a decrease in *bcl-2* and *bax* mRNA expression. It should be noted that the ethanol extract showed the ability to stop the cell cycle in G0/G1 and in phase S.

Natural extracts of the *Eugenia involucrate* DC fruits and seeds obtained using ethanol (99.4%) were tested for their antitumor activity. Only the seed extracts showed a cytotoxic effect, with an IC_50_ of 645 µg/mL in the PANC-1 pancreatic cell line, showing the ability to induce apoptosis through the alteration of the mitochondrial membrane (intrinsic pathway). Alteration in the structure of the cytoskeleton and an increase in the production of reactive oxygen species (ROS) were also observed in this cell line [28]. *Cleistocalyx operculatus* (Roxb.) shoots were used to extract the biocomposite 2′,4′-dihydroxy-6′-methoxy-3′, 5′-dimethylchalcone (DMC) by using 70% ethanol. This compound showed an IC_50_ of 10.5 and 12.2 µM in PANC-1 and MIA PaCa-2 pancreatic cell lines, respectively. A significant increase in caspase-3 and -9 and a greater inhibition of *bcl-2* suggested an apoptosis mediated by the mitochondrial pathway [29]. In addition, ethanol (70%) and distilled water extracts from fresh leaves of three species of Eucalyptus (*E. robusta Sm*, *E. microcorys F. Muell* and *E. saligna Sm*) were analyzed. Only the extracts of the last two species showed antitumor activity. *E. microcorys* leaf extract showed an IC_50_ between 68 µg/mL (aqueous extract) and 76 µg/mL (70% ethanol extract) in MIA PaCa-2 pancreatic cell line. Furthermore, distilled water extract stimulated the activity of caspase-3/7, suggesting induction of cell death by apoptosis. In *E. saligna*, only the ethanol extract showed activity (IC_50_ of 53 µg/mL) [30].

On the other hand, *Gloriosa superba* L. seeds, with high levels of colchicine (GS), were used to generate extracts of rich and poor GS. The first extract showed high cytotoxicity in PANC-1 and Panc02 cells (IC_50_ of 0.45 and 0.17 µg/mL, respectively). By contrast, the second extract (designated GS2B) showed an IC_50_ of 9.49 µg/mL in Panc02 cells (50 times higher). *In vivo* assays of both extracts using Panc02 tumors showed a reduction in tumor volume with a decrease in *ki67* expression and an increase in apoptosis-mediated caspase-3 [31]. Moreover, an extract called BAEE was generated using absolute ethanol extraction of *Prunus armeniaca* L. *kernels* which was tested in PANC-1 cells showing an IC_50_ of 704 µg/mL with an increase in *bax/bcl-2* ratio and an overexpression of caspase-3 [32].

As shown in Table 2, extracts from *C. nutans* stems and leaves were generated using methanol (polar compounds), hexane, and diethyl ether (nonpolar compounds). The results showed that only the nonpolar extracts had antitumor activity, with an IC_50_ ranging between 30.91 and 39.12 µg/mL in the three pancreatic tumor lines tested (AsPC1, BxPC3 and SW1990) This extract showed synergistic action in combination with gemcitabine, increasing the expression of proapoptotic proteins (BAX and BID) and decreasing the expression of antiapoptotic proteins (BCL-2, XIAP, CIAP-2) [33]. In addition, a 70% methanol extraction of *C. cordifolioidea* roots was used to isolate the cordifoliketonas A biomolecule. This compound showed an IC_50_ ranging from 4.18 to 5.56 µg/mL in pancreatic tumor cell lines. AsPC-1, BxPC-3 and PANC-1 produced apoptosis through overexpression of proapoptotic proteins (BAX, BAD) and decreased expression of antiapoptotic proteins (BCL-2 and BCL-XL). An in vivo assay showed similar activity, decreasing tumor growth compared to controls [34]. In addition, the monogalactosyl diacylglycerol (MGDG) molecule was isolated from a 70% ethanolic extract of the species *S. oleracea*. This molecule was tested in vitro (MIA PaCa-2, BxPC-3, AsPC-1 and PANC-1 cell lines), obtaining an IC_50_ between 18.5 and 26.9 µg/mL through apoptosis associated with the overexpression of PARP, caspase-3 and BAX (proapoptotic proteins). The in vivo assay performed on MIA PaCa-2 tumors showed that MGDG had synergy with irradiation [35]. Another study analyzed the activity of a flavonoid extract (TFAE) from dried roots of *S. baicalensis* obtained in ethyl acetate. The in vitro assay showed cytotoxic activity on pancreatic cell lines BxPC-3 and PANC-1, with IC_50_ ranging from 6.5 to 41.7 and 8.9 to 47.4 µg/mL, respectively. TFAE had no effect on the nontumor cell line HPDE6-C7. This extract produced cell toxicity through the induction of autophagy (via PI3K/AKT/mTOR). Its antitumor activity was also confirmed in an in vivo BxPC-3 model decreasing tumor growth [36].

### 3.2. Plant Species and Isolated Compounds That Induce Cell Death through Alteration of Pathways Activated by KRAS Mutation

Table 3 shows plant species and isolated compounds that induce cell death through alteration of pathways activated by KRAS mutation. In fact, *T. officinale* roots were used to generate a cytotoxic aqueous extract against BxPC-3 and PANC-1 pancreatic cell lines (IC_50_, 5 mg/mL for both cell lines). The cytotoxic mechanism was mediated by autophagy with an increase in LC3-II levels. However, a high expression of caspase-8 was also detected [37]. In addition, grandifloracin (GF), a molecule obtained from the *U. dac* stem (methylene chloride extract), showed activity against the PANC-1 cell line (IC_50_ 14.5 µM) through the inhibition of the AKT/mTOR pathway, key in the start of the autophagy process [38].

On the other hand, nine compounds from *V. anthelmintica* dried fruits were extracted using 80% ethanol. Only isorhamnetin and luteolin showed antitumor activity against PANC-1 (IC_50_, 19.6 µM and 18.1 µM, respectively). Isorhamnetin induced inhibition of the RAS/MAPK pathway and cell cycle arrest in the S phase [39]. In another study, eight molecules were isolated from *C. hystix* fruit extracts. Bergamottin was the most interesting molecule, showing an IC_50_ of 4.6, 2.2 and 9.4 µM in PANC1, MIA PaCa-2 and PSN-1, respectively. Its cytotoxic activity was due to the induction of cell death by autophagy [40].

Finally, the EEIHL extract obtained from *I. helenium* (trizoma and dried root) using 95% ethanol induced an IC_50_ of 4.3 µg/mL in CFPAC-1 pancreatic cells. This extract induced autophagy, apoptosis (with increased proapoptotic proteins and decreased antiapoptotic proteins) and cell cycle arrest in G0/G1 phase. Furthermore, EEIHL inhibited cell migration through increased *e-cadherin* expression [41].

### 3.3. Plant Species and Isolated Compounds That Induce Cell Death through Arrest in Some Phase of the Cell Cycle

A relevant group of isolated compounds was able to induce cell death through arrest in some phases of the cell cycle, as shown in Table 4. The stems of *H. hirsute* were processed with 40% methanol to obtain a saponin-enriched extract. HPLC allowed separation of six fractions (F0–F5). Apart from F0, all fractions showed an IC_50_ between 3.11–7.76 μg/mL and 3.71–17.12 μg/mL for MIA PaCa-2 and BxPC-3, respectively. The mechanism of action of F2, F3, F4 and F5 was cell cycle arrest in the S phase [42]. On the other hand, two extracts using 100% ethanol (EEOL) and water (AEOL) were obtained from *Ocimum sanctum* L. dried leaves. EEOL was tested on AsPC-1 and MIA PaCa-2 cell lines, showing an IC_50_ of 46 and 69 µg/mL, respectively, and a significant arrest of the cell cycle in G2/S. AEOL was tested in an AsPC-1 in vivo model, showing a reduction in antiapoptotic protein expression and increasing expression of BAD (proapoptotic) and E-cadherin [43]. In another study, an ethanolic extract of *P. acerifolium* bark (PaEBE) was tested on PANC-1 cells, showing an IC_50_ of 74.22 µg/mL and intense cell cycle arrest in the sub-G1 phase accompanied by an increased ROS production and altered mitochondrial membrane potential [44].

The association of extracts has also been assayed to determine the antitumor potential in PC. A 40% ethanolic extract of the combination of *Meliae* fruit, *Cinnamon* bark and *Sparganium rhizome* (named H39) showed a significant antitumor effect on PC cells PANC-1 alone (IC_50_ 0.07 mg/mL) or in combination with gemcitabine (3 nM) (IC_50_ of 0.05 mg/mL). H39 induced cell death by arresting the cell cycle at the G0/G1 phase. This extract was able to decrease cell migration and the expression of antiapoptotic genes such as *JAK2* and *XIAP* [45]. In some cases, such as *O. horridus* (root bark), ethanolic (70%) extract was associated to gemcitabine, cisplatin and paclitaxel against the PANC-1 cell line in 2D and 3D conditions, showing that the extract significantly enhanced the antiproliferative activity of both gemcitabine and cisplatin at some concentrations. The cell death mechanism described was a cell cycle arrest in the S phase [46].

Aqueous extraction (distilled water) has also been used as in the case of *E. microcorys* and *M. oleifera* leaves. In the first case, five fractions (F1–F5) were isolated after HPLC, but only F1 showed antiproliferative potential in the MIA PaCa-2 cell line (IC_50_ 93.11 μg/mL) through G2/M cell cycle arrest [47]. In the second case, the extract was tested in three different pancreatic adenocarcinoma cell lines (PANC-1, COLO 357 and p34) showing IC_50_ between 1.1 and 1.8 mg/mL. The cytotoxicity was caused by the cell cycle arrest in the sub-G1 phase showing synergistic activity with cisplatin [48].

### 3.4. Plant Species and Isolated Compounds That Induce Cell Death through the Alteration of Other Important Factors in the Formation of Pancreas Cancer

Some plant species and isolated compounds induced cell death through the alteration of other important factors in the etiology of pancreas cancer (Table 5). Tumor cells are capable of properly altering the tumor microenvironment, generating an environment in which they have greater complications for cell proliferation. Natural compounds that can alter these environments in pancreatic adenocarcinoma cell lines were analyzed. In this context, two natural compounds inhibited VEGF retarding tumor growth: SB365 and SH003. SB365 is a saponin D from roots of *P. koreana* (50% ethanol extraction), which demonstrated high antitumor activity in five different pancreatic cell lines (MIA PaCa-2, BxPC-3, PANC-1, AsPC-1 and HPAC, IC_50_ between 0.8 and 2 µM). Furthermore, this molecule inhibited both HIF-1α and VEGF, two potent inducers of angiogenesis and was capable of inducing apoptosis by the intrinsic pathway [49]. SH003 is a 30% ethanolic extract obtained from three plants (*A. membranaceus*, *A. gigas* and *T. Kirilowii*), which was tested on endothelial cells (HUVECs) observing a reduction in angiogenesis (inhibiting VEGF phosphorylation) and a delay in tumor growth derived from the Panc-28-LUC cell line [50]. In addition, another article used an extraction process with CO2 and ethanol to produce an extract from *A. millefolium* called Yarrow SFE, which showed an IC_50_ of 31.45 µg/mL on MIA PaCa-2 cells. Its activity was related to the alteration of lipid homeostasis, showing a decrease in the expression of *SREBF1*, *FASN* and *SCD*. These genes are overexpressed in PC and are key factors for fatty acid production. *In vivo*, this extract produced a significant tumor growth inhibition, a decrease in *KI67*-positive cells and a downregulation in *SREBF1* [51]. Lastly, a 70% ethanolic extract from dried spinach of the species *S. oleracea* allowed the isolation of the monogalactosyl diacylglycerol (MGDG). This compound induced an IC_50_ of 22, 15.1 and 18.8 nM in PANC-1, BxPC-3 and MIA PaCa-2, respectively and exerted its antitumor activity through the selective inhibition of polymerases α, δ and ε and inhibition of DNA replication [52].

## 4. Discussion

Both the extracts and the biomolecules analyzed in this review were characterized by showing significant antitumor activity in vitro against different PC cell lines through several molecular pathways.Most of the analyzed extracts were obtained using polar solvents, generally ethanol. Therefore, most of the extracted compounds with biological activity against PC were phenols and flavonoids with high affinity for these solvents [53]. Other polar solvents, such as methanol or water, were also used. It is known that the extracts that use ethanol are usually the most efficient for the extraction of polyphenols [54], although they are characterized by their high cytotoxicity. Then, in the study on *O. horridus*, it was illustrated how the extracts obtained from the stem of the plant using ethanol showed a higher IC_50_ than those obtained with distilled water [26]. Likewise, the study on *C. nutans* showed the relevance of the extraction method and the part of the plant used [33]. However, the extraction of bioactive compounds in plants needs to be further investigated, since factors such as solvent temperature are critical to optimize the process. Thus, a study that tried to extract bioactive compounds in methanol found that the highest biological activity was exerted in those extracts carried out at 40 °C [55].

In addition, various studies used these plant products together with current antitumor drugs. Indeed, a synergistic effect between gemcitabine and extracts and/or molecules of *C. nutans*, *E. microcorys* and *S. oleracea* was described. *M. oleifera* extract showed greater activity in combination with platins. It should be remembered that the FOLFIRINOX regimen contains oxaliplatin among its drugs [33,47,48,52]. Synergies between plant extracts and chemotherapeutic drugs may be related to the ability of natural compounds to evade drug resistance inhibiting drug efflux pumps (such as p-glycoprotein) and increasing their internalization or decreasing drug modification by cytochromes or detoxifying enzymes (i.e., glutathione transferases) [56]. Furthermore, the introduction of plant extracts may have a second function, such as reducing systemic chemotherapeutic toxicity. Plant extracts are known to be generally well tolerated and generally nontoxic in models of healthy cells in two-dimensional culture [57]. In addition, in recent years, progress has been made to reduce the therapeutic dose and improve its ability to target tumor cells, mainly using nanoparticles loaded with compounds isolated from extracts [58,59,60,61,62]. On the other hand, it has been shown that these natural products are capable of reducing the side effects of chemotherapy and radiotherapy and the systemic toxicity derived from aggressive treatments [41]. Most of the articles included in this systematic review (39%) showed that the plant extracts generated cell death through the apoptotic pathway. Cycle arrests that also end up leading to this programmed cell death pathway were also described. This coincides with the literature where the majority of plant extracts trigger cytotoxicity by inducing cell death through a route of apoptosis [63,64,65].

Of the 28 included studies, only eight analyzed in vivo effects of these drugs. The study on *S. baicalensis* being worth noting since not only was a reduction in tumor size demonstrated but also an in vitro absence of effect on HPDE6-C7 nontumor cells [36]. These encouraging results in mice mean that some plant extracts or compounds derived from them are capable of reaching advanced phases of clinical trials. In this context, curcumin was tested in various clinical trials both alone and in combination with antitumor drugs such as FOLFOX. The results showed that supplementation of FOLFOX therapy with a daily dose of curcumin was tolerable in patients with metastatic CRC, increasing survival with no significant difference in patient quality of life or neurotoxicity [66]. Meanwhile, another clinical trial using curcumin (8 g/day) with gemcitabine in patients with pancreatic metastases demonstrated a significant response, although some patients (29%) showed high gastrointestinal toxicity [67]. On the other hand, it should be noted that natural compounds (Table S1) are isolated in small amounts, which prevents comprehensive drug screening in vivo, being necessary the role of organic synthesis for the total synthesis of natural compounds or for the synthesis of their simplified and bioactive analogs. This entails advantages over native compounds, such as shelf life, improved biological parameters or being able to conjugate them with antibodies or drugs [68,69]. In this context, Casertano et al. chemically manipulated a natural metabolite of marine origin (phospheleganine) to optimize its potential as an antidiabetic and to obtain a higher yield [70].

Finally, it is worth noting the analysis that some researchers carried out on the hypoxic and nutrient-deficient microenvironment of PC, which could reduce the antitumor drug effects. Consequently, the effect of plant extracts and molecules on tumor cells cultured in nutrient-rich and nutrient-poor media was tested. In fact, Ueda et al. [38] showed how grandiflorazine obtained from *U. dac* showed high antitumor activity against cells in nutrient-poor environments and had no effect on cells in more favorable environments and that some antitumor drugs lacked activity in this adverse environment. These results are consistent with other studies in which plant extracts generated high cytotoxicity under conditions of low nutrient content [71,72,73,74]. This may be because tumor cells, including PC cells, showed high vulnerability under low nutrient conditions when the inhibition of the redox system occurred. Considering that most plant extracts possess antioxidant activity, this phenomenon could explain the observed effect [75].

## 5. Conclusions

Plant extracts are presented as an opportunity to improve the treatment of PC and to obtain bioactive molecules that implement novel strategies to address this type of tumor. Various mechanisms of cell death related to apoptosis, KRAS mutation and cell cycle modulation have been described when extract or isolated biomolecules have been used in pancreatic tumor culture cells. *In vitro* studies have been a first step to determine biomolecules that guarantee an effect on these tumor cells. Although more studies will be necessary to corroborate its activity, the results obtained to date will make it possible to analyze other parts of the plant, to improve extraction methods and to develop more complex tests improving pancreatic cancer therapy. It will be necessary to increase the number of tests in nontumor cells, the in vivo assays and the toxicity studies to take the step of carrying out clinical trials that allow them to be used as adjuvant therapy in the future. The role of organic synthesis would provide improvements in these isolated compounds of plant origin.

## Figures and Tables

**Table 1:** Summary of the articles included indicating the mechanism of action by which the extracts or compounds isolated from different plant species act.

Family	Species	N° Article	Part of Plant	Mechanism of Action
Araliaceae	*Oplopanax horridus* (Sm.) Miq.	3	Stem; Root	Apoptosis
Myrtaceae	*Eugenia involucrata* DC.	1	Fruits; Seed	Apoptosis
	*Cleistocalyx operculatus* (Roxb.)	1	Bud	
	*Eucalyptus robusta* Sm., *Eucalyptus microcorys* F.Muell. and *Eucalyptus saligna* Sm.	2	Leaves	
	*Syzygium aromaticum* L.	1	Entire plant	KRAS mutation
Acanthaceae	*Clinacanthus nutans* (Burm.f.) Lindau	1	Leaves Stem	Apoptosis
Rosaceae	*Prunus armeniaca* L.	1	Seed	Apoptosis
Campanulaceae	*Codonopsis cordifolioidea* P.C. Tsoong	1	Root	Apoptosis
Amaranthaceae	*Spinacia oleracea* L.	2	Leaves	Apoptosis
Rutaceae	Citrus hystrix DC.	1	Fruit	KRAS mutation
Annonaceae	*Uvaria dac*	1	Stem	KRAS mutation
Colchicaceae	*Gloriosa superba* L.	1	Seed	Apoptosis
Lamiaceae	*Scutellaria baicalensis* Georgi	1	Root	Apoptosis
	*Ocimum sanctum* L.	1	Leaves	Arrest cell cycle
Asteraceae	*Taraxacum officinale* L.	1	Root	KRAS mutation
	*Vernonia anthelmintica* L.	1	Fruit	
	*Inula helenium* L.	1	Rhizome Root	
Malvaceae	*Helicteres hirsuta* Lour.	1	Stem	Arrest cell cycle
	*Pterospermum acerifolium* L.	1	Bark	
Moringaceae	*Moringa oleífera* Lam.	1	Leaves	Arrest cell cycle
Ranunculaceae	*Pulsatilla koreana* (Yabe ex Nakai)	1	Root	Other alterations
Fabaceae, Apiáceas, Cucrbitaceae	*Astragalus membranaceus* (Fisch.), *Angelica gigas* Nakai, *Trichosanthes Kirilowii* Maxim.	1	Entire plant	Other alterations
Meliaceae, Lauraceae	Meliae, Cinnamon, Sparganium	1	Fruit; Bark; Rhizome	Arrest cell cycle
Asteraceae	*Achillea millefolium* L.	1	Entire plant	Other alterations

**Table 2:** Plant extract and isolated compounds that induce in vitro cell death in pancreatic cell lines by apoptosis.

Material (Reference)	Extraction Method	Isolated Compounds	Cell Line	IC_50_	Mechanism of Action
Stem of *Oplopanax* *Horridus* (Sm.) Miq. [26]	Ethanol 70% for DCEE Ddw for DCWE	DCEE DCWE DCA (1–4)	PANC-1 BxPC-3	DCEE: PANC-1: 5.5–5.8 µg/mL BxPC-3: 21–34 µg/mL DCWE: PANC-1: 4950 µg/mL DCA: PANC-1: 0.22–1.593 µg/mL BxPC-3: 0.82–1.404 µg/ml	DCEE and DCWE induced apoptosis and nuclear necrosis. DCA induced apoptosis by both the intrinsic and extrinsic pathways.
Dried root of *Oplopanax horridus (Sm.) Miq.* [27]	Ethanol 70%	DC DCA	PANC-1 BxPC-3	DC: PANC-1: 0.0058% (*v*/*v*) BxPC-3: 0.021% (*v*/*v*) DCA: PANC-1: 0.73 µM BxPC-3: 2.71 µM	DC caused cell cycle arrest and apoptosis induction, increasing caspase-3 expression. DC and DCA decreased expression of *BCL-2* and *BAX* mRNA.
Fruits and seeds of *Eugenia involucrata* DC. [28]	Ethanol 99.4%	FE SE	PANC-1	SE: PANC-1: 645 µg/mL	SE caused apoptosis induction and increased ROS generation.
Bud of *Cleistocalyx operculatus* (Roxb.) [29]	Ethanol 70%	DMC	PANC-1 MIA PaCa-2	DMC: PANC-1: 10.5 µM MIA PaCa-2: 12.2 µM	DMC produced apoptosis by increasing the activity of caspase-3 and -9 and inhibiting the expression of antiapoptotic protein such as BCL-2.
Fresh leaves of *Eucalyptus robusta Sm.*, *E. microcorys* F.Muell and *E. saligna* Sm. [30]	Ethanol 70% Ddw	Ethanol and Ddw extract of both	MIA PaCa-2 BxPC-3 CFPAC-1 HPDE	Ethanol extract and ddw extract of *E. microcorys*: MIA PaCa-2: 64.66–86.05 µg/mL Ethanol extract of *E. saligna*: MIA PaCa-2: 115.52 µg/mL	Ddw extract of E.microcorys induce apoptosis through caspase-3/7 expression
Dried sedes of *Gloriosa superba* L. [31]	Ethanol 80%	GS GS2B	PANC-1 Panc02	GS: PANC-1: 0.45–0.59 µg/mL Panc02: 0.17–0.19 µg/mL GS2B: Panc02: 9.49 µg/mL	In a Panc02 in vivo model, both extract increased caspase-3 levels in tumor cells and decreased *ki67* expression
Leaves and stems of *Clinacanthus nutans* (Burm.f.) [33]	Methanol and dichloromethane for polar compounds Hexane and diethyl ether for nonpolars	LP LN SP SN	AsPC-1 BxPC-3 SW1990	SN: AsPC-1: 31.21 µg/mL BxPC-3: 39.12 µg/mL SW1990: 30.91 µg/ml	Synergistic action with GMZ, producing an increase in proapoptotic proteins such as BAX and a decrease in antiapoptotic proteins such as BCL-2, XIAP and CIAP-2.
Roots of *Codonopsis cordifolioidea* P.C. Tsoong [34]	Methanol 70%	Cordifoliketones A	BxPC-3 PANC-1 AsPC-1	Cordifoliketones A: AsPC-1: 5.56 µg/mL BxPC-3: 4.26 µg/mL PANC-1: 4.18 µg/mL	Apoptosis induction by increased expression of proapoptotic proteins (BAX, BAD) and decreased expression of antiapoptotic proteins (BCL-2, BCL-XL). Cell migration inhibition and decreased in vivo tumor size.
Seeds of *Prunus armeniaca* L. [32]	Ethanol	BAEE	PANC-1	BAEE: PANC-1: 704 µg/mL	Apoptosis induction by increased BAX and caspase-3 expression and BCL-2 inhibition.
Dried leaves of *Spinacia oleracea* L. [35]	Ethanol 70%	MGDG	MIA PaCa-2 PANC-1 AsPC-1 BxPC-3	MGDG: MIA PaCa-2: 18.5 µM PANC-1: 25.6 µM AsPC-1: 22.7 µM BxPC-3: 26.9 µM	Apoptosis induction observed in MIA PaCa-2 cell line by increased cytochrome C levels in the cytosol, increased expression of PARP, caspase-3 and BAX and decreased expression of BCL-2 (antiapoptotic protein). Potentiation of the suppressive effects of radiation, both in MIA PaCa-2 in vitro and in vivo model.
Dried root of *Scutellaria baicalensis* Georgi [36]	Ethyl acetate	TFAE	BxPC-3 PANC-1 HPDE6c7	TFAE: BxPC-3: 41.7 µg/mL (24 h) 12.3 µg/mL (48 h) 6.5 µg/mL (72 h) PANC-1: 47.4 µg/mL (24 h) 20.5 µg/mL (48 h) 8.9 µg/mL (72 h)	Induction of apoptosis by caspase-3/8, PARP and BID in BxPC-3, without action on BCL-2. Induction of autophagy, visible in increased LC3 II, through decreased activity in the PI3K/AKT/mTOR pathway. Tumor growth decreased and absence of healthy cells toxicity.

BAEE (Bitter Apricot Ethanolic Extract); DC (ethanolic extract of dried root of O. horridus); DCA (Devil’s Club Falcarinol-Type Polyacetylenes); DCEE (Devil’s club ethanol extract); DCWE (Devil’s club aqueous extract); Ddw (distilled water); DMC (2′,4′-dihydroxy-6′-methoxy-3′,5′-dimethylchalcone); FE (fruit extract); GS (extract of G. superba); GS2B (extract of G. superba poor in colchicine and rich in colchicosides); HPLC (high-performance liquid chromatography); IC_50_ (half-maximal inhibitory concentration); LN (nonpolar extract from leaves); LP (polar extract from leaves); MGDG (monogalactosil diacilglicerol); PARP (poly (ADP-ribose) polymerase); ROS (reactive oxygen species); SE (seed extract); SN (nonpolar extract from stem); SP (polar extract from stem); TFAE (total flavonoids aglycone extract).

**Table 3:** Plant extract and isolated compounds that induce cell death through the alteration of the pathways activated by the KRAS mutation.

Material (Reference)	Extraction Method	Isolated Compounds	Cell Line	IC_50_	Mechanism of Action
Root of *Taraxacum officinale* L. [37]	Ddw	DRE	BxPC-3 PANC-1	DRE: 5 mg/mL in both cell lines.	Induction of autophagy. Apoptosis induction through destabilization of the mitochondrial membrane, causing the release of proapoptotic factors and increased caspase-8 activity.
Stem of *Uvaria dac* [38]	CH_2_Cl_2_	GF	PANC-1	GF: PANC-1: 14.5 µM	Increased cytotoxicity in PANC-1 cells grown in nutrient-free medium and AKT/mTOR inhibition.
Dried fruits of *Vernonia anthelmintica* L. [39]	Ethanol 80%	-Eriodictyol -Apigenin -Butein -Butin -Isorhamnetin -Sulfuretin -Luteolin -3,5-O-DCAME -3,4-O-DCAME	PANC-1	Isorhamnetin: PANC-1: 19.6 µM Luteolin: PANC-1: 18.1 µM	Isorhamnetin produced S-phase arrest of tumor cells, inhibition of the RAS/MAPK pathway through inhibition of MEK phosphorylation and suppression of in vitro cell migration.
Fruits of *Citrus hystrix* DC. [40]	Ethanol 70%	-(R)-(+)-OM -(R)-(+)-OHI -(S)-(−)-O -(R)-(+)-P -Bergamottin -(R)-(+)-6HMBD -7-hydroxycoumarin	PANC-1 MIA PaCa-2 PSN-1	Bergamottin: PANC-1: 4.6 µM MIA PaCa-2: 2.2 µM PSN-1: 9.4 µM	Bergamottin produced selective cytotoxicity on cells in a poor-nutrient medium, inhibited AKT expression and cell migration
Rhizome and dried roots of *Inula helenium* L. [41]	Ethanol 95%	EEIHL	CFPAC-1	EEIHL: CFPAC-1: 4.3 µg/mL	Cell cycle arrest in G0/G1 phase, depolarization of membrane potential, apoptosis induction, inhibition of AKT and STAT-3 phosphorylation and cell migration inhibition by augmented expression of E-cadherin.

CH_2_Cl_2_ (dichloromethane); 3,4-O-DCAME (3,4-O-dicaffeoylquinic acid methyl ester); 3,5-O-DCAME (3,5-O-dicaffeoylquinic acid methyl ester); Ddw (distilled water); DRE (dried root extract); EEIHL (*Inula helenium* L. ethyl acetate extract); GF (grandifloracin); half-maximal inhibitory concentration (IC_50_); pRB (retinoblastoma protein); (R)-(+)-OHI ((R)-(+)-oxypeucedanin hydrate isoimperatorin); (R)-(+)-OM ((R)-(+)-oxypeucedanin methanolate); (R)-(+)-P ((R)-(+)-pabulenol); (R)-(+)-6HMBD (R)-(+)-6′-hydroxy-7′-methoxybergamottin -(R)-(+)-6′, 7′-dihydroxybergamottin (S)-(−)-O ((S)-(−)-oxypeucedanin).

**Table 4:** Plant extract and isolated compounds that induce cell death through arrest in some phase of the cell cycle.

Material (Reference)	Extraction Method	Isolated Compounds	Cell Line	IC_50_	Mechanism of Action
Stem of *Helicteres hirsuta* Lour. [42]	Methanol 40%	Extract enriched in saponin. 6 fractions were obtained from the extract (F0–F5) by HPLC.	MIA PaCa-2 BxPC-3	F1: MiapaCa-2: 7.76 µg/mL BxPC-3: 17.12 µg/mL F2: MiapaCa-2: 4.54 µg/mL BxPC-3: 9.25 µg/mL F3: MiapaCa-2: 3.85 µg/mL BxPC-3: 3.71 µg/mL F4: MiapaCa-2: 3.88 µg/mL BxPc-3: 5.16 µg/mL F5: MiapaCa-2: 3.11 µg/mL BxPC-3: 4.23 µg/mL	Fractions F2, F3, F4, F5 produced cell cycle arrest in phase S.
Dried leaves of *Ocimum sanctum* L. (Holy Basil) [43]	Ethanol 100%	EEOL AEOL	AsPC-1 MIA PaCa-2	EEOL: AsPC-1: 46 µg/mL MiapaCa-2: 69 µg/mL	Cell cycle arrest in G2/S in MIA PaCa-2 cells Decreased expression of NF-κB, increased expression of proapoptotic protein Bad. Decreased expression of BCL-2 and BCL-XL, in addition to increasing of BAD and E-cadherin in in vivo conditions.
Bark of *Pterospermum acerifolium* L. [44]	Ethanol 70%	PaEBE	PANC-1	PaEBE: PANC-1: 74.22 µg/mL	Cell cycle arrest in G1 phase, increased ROS production and alteration of mitochondrial membrane, inducing apoptosis.
Combination of *Meliae fructus*, *Cinnamon bark*, *Sparganium rhizome* [45]	Ethanol 40%	H3	PANC-1	H3: PANC-1: 0.07 mg/mL	Cell cycle arrest in G0/G1 phase, cell migration inhibition, decreased expression in mRNA expression of genes associated with apoptosis (*JAK2*, *CXCR4*, *XIAP*) and increased cytochrome C levels in the cytoplasm.
Leaves of *Eucalyptus microcorys* F.Muell [47]	Ddw	Fractions (F1–F5)	MIA PaCa-2 BxPC-3 CFPAC-1	F1: MIA PaCa-2: 93.11 µg/mL	Induced cell cycle arrest in G2/M phase, higher in combination with GMZ and apoptosis induction through decreased BCL-2 and increased BAX expression.
Dried root of *Oplopanax horridus* (Sm.) [46]	Ethanol 70%	DC DCA	PANC-1	DC: 2D culture: 1/(17200) dilution 3D culture: 1/(3311) dilution DCA: 2D culture: 0.73 µM 3D culture: 3.15 µM	Cell cycle arrest observed in S phase in 3D cultured cells.
Leaves of *Moringa oleífera* Lam. [48]	Ddw	Aqueous extract	PANC-1 COLO 357 p34	PANC-1: 1.1 mg/mL COLO 357: 1.8 mg/mL p34: 1.5 mg/mL	Increased number of PANC-1 cells in sub-G1 phase, decreased expression of proteins of the NF-kB signaling pathway (p65, IkBa) and possible synergistic action in combination with cisplatin.

AEOL (aqueous extract of leaves of *O. sanctum*); DC (ethanol extract); DCA (Devil’s Club Falcarinol-Type Polyacetylenes); Ddw (distilled water); EEOL (ethanol extract of leaves of *O. sanctum*); H3 (extract of plant combination); HPLC (high-performance liquid chromatography); IC_50_ (half-maximal inhibitory concentration); PaEBE (ethanol extract from bark of *Pterospermum acerifolium* (L.)); ROS (reactive oxygen species).

**Table 5:** Plant extract and isolated compounds that induce cell death through the alteration of other important factors in the formation of pancreatic cancer.

Material (Reference)	Extraction Method	Isolated Compounds	Cell Line	IC_50_	Mechanism of Action
Root of *Pulsatilla koreana (Yabe ex Nakai)* [49]	Ethanol 50%	SB365	PANC-1 MIA PaCa-2 BxPC-3 AsPC-1	SB365: 0.8–2 µM in all cell lines	Inhibition of the expression of HIF-1α and VEGF in a hypoxic environment with antiangiogenic effects also in mice, increased cytosolic cytochrome C and caspase-3 and decreased BCL-2 levels. Decreased number of KI67-positive cells.
*Astragalus membranaceus*, *Angelica gigas* and *Trichosanthes Kirilowii* Maximowicz [50]	Ethanol 30%	SH003	HUVECs	SH003: 0.23–2.67 µg/mL	Inhibits VEGF/VEGFR-2-mediated angiogenesis in vitro, retarding the growth of Panc-28-LUC cells in mice. Reduction of KI67, p-VEGFR2 and MMP-9 levels and increased levels of caspase-3.
*Achillea millefolium* (Yarrow) [51]	CO_2_	Yarrow SFE	MIA PaCa-2 PANC-1	Yarrow SFE: MIA PaCa-2: 31.45 µg/mL	Decreased expression of SREBF1, FASN and SCD, involved in the production of fatty acids (overexpressed in PANC-1 and MIA PaCa-2 cells). Inhibition of in vivo tumor growth.
*Spinacia oleracea* L. [52]	Ethanol 70%	MGDG	PANC-1 BxPC-3 MIA PaCa-2	MGDG: PANC-1: 22 nM BxPC-3: 15.1 nM MIA PaCa-2: 18.8 nM	Selective inhibition of α, δ and ε polymerases (with IC50 of 10.7–22 µM) and gamma, with IC_50_ of 35.1 µM. Induction of apoptosis on MIA PaCa-2.

CO_2_ (carbon dioxide); Ddw (distilled water); IC_50_ (half-maximal inhibitory concentration; MGDG (monogalactosil diacilglicerol); SB365 (*Pulsatilla* saponin D); SFE (supercritical fluid extraction); SH003 (extract composed of *Astragalus membranaceus*, *Angelica gigas* and *Trichosanthes Kirilowii Maximow*).

## Data Availability

Not applicable.

## Supplementary Materials

The following supporting information can be downloaded at: https://www.mdpi.com/article/10.3390/life12111765/s1, Table S1: Chemical structure of the compounds isolated in the articles included in the systematic review.
