# Supplementary material for: Active Biomolecules from Vegetable Extracts with Antitumoral Activity against Pancreas Cancer: A Systematic Review (2011–2021)
Source: Life (Basel). 2022 Nov 2;12(11):1765. doi: 10.3390/life12111765 (PMC9695035; doi:10.3390/life12111765)
Supplement: Supplementary file 1 [file life-12-01765-s001.zip › life-1973221-supplementary.pdf]

Supplemental material: Active biomolecules from vegetable extracts with antitumoral activity against pancreas cancer: a systematic review (2011-2021)

Table S1. Chemical structure of the compounds isolated in the articles included in the systematic review.

Material (Reference)	Isolated Compounds	Chemical structure	Mechanism of action
[14,15]	DCA	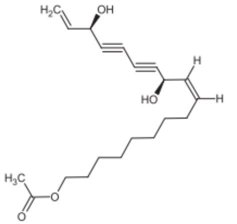	Apoptosis
[17]	DMC	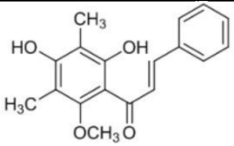	
[19]	GS and GS2B rich in **(A)** colchicoside, **(B)** 3-O-demethylcolchicine and **(C)** colchicine	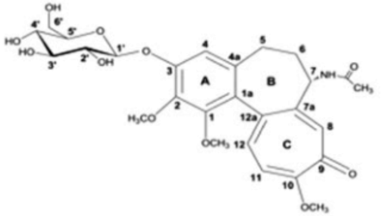 A	

		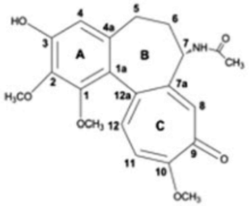<br>B	
		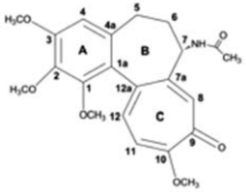<br>C	
[22]	Cordifoliketones A	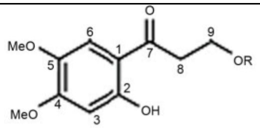	
[20]	BAEE rich in amygdalin	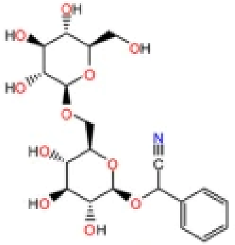	
[23]	MGDG	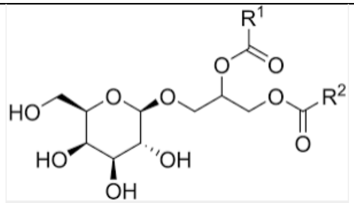	

[26]	GF	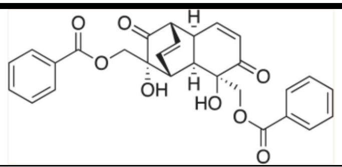	KRAS mutation
[27]	(1) Eriodictyol<br>(2) Apigenin<br>(3) Butein<br>(4) Butin<br>(5) Isorhamnetin<br>(6) Sulphuretin<br>(7) Luteolin<br>(8) 3,5-O-DCAME<br>(9) 3,4-O-DCAME	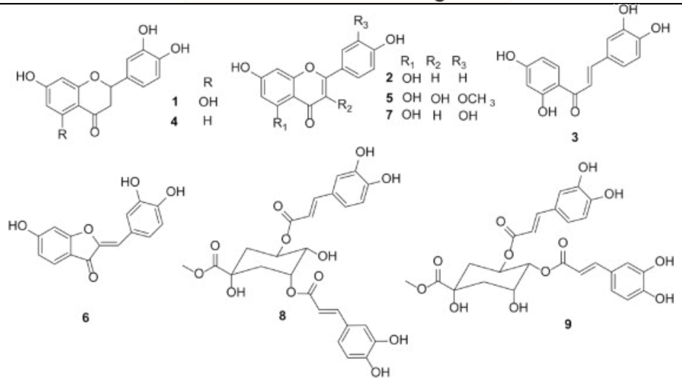	
[28]	Bergamottin	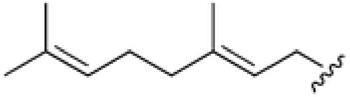	
[34]	DCAT	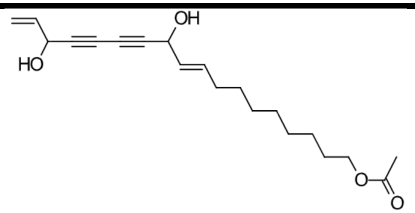	Arrest in some phase of the cell cycle

[37]	SB365	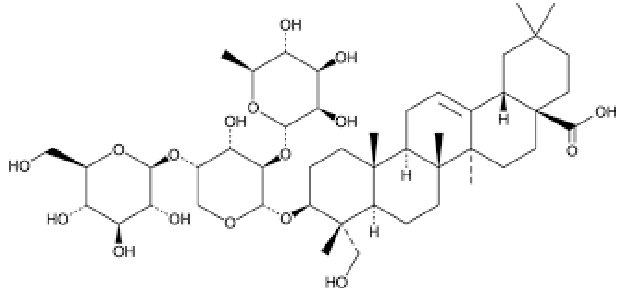	Alteration of other important factors
[40]	MGDG	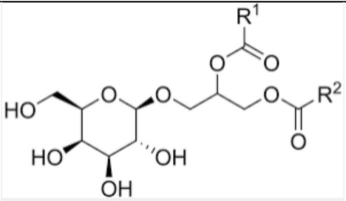	

DCA (Devil’s Club Falcarinol-Type Polyacetylenes); DCAT (9,17-octadecadiene-12,14-diyne-1,11,16-triol 1) DMC (2 ‘, 4’-Dihydroxy-6’-methoxy-3’, 5’-dimethylchalcone); GF (Grandifloracin); GS (*Gloriosa superba* extract); GS2B (*Gloriosa superba* second extract); MGDG (monogalactosyl diacylglycerol); 3,4-O-DCAME (3,4-O-dicaffeoylquinic acid methyl ester); 3,5-O-DCAME (3,5-O-dicaffeoylquinic acid methyl ester); SB365 ((Pulsatilla saponin D)
